# Geotechnical Assessment of Foundation Stability for Preserving the Agrippa Monument at the Acropolis of Athens

**DOI:** 10.3390/s25010219

**Published:** 2025-01-02

**Authors:** Vassilis Marinos, Georgios Prountzopoulos, Dimitra Papouli, Dionisia Michalopoulou, Vassiliki Eleftheriou

**Affiliations:** 1 School of Civil Engineering, Geotechnical Division, National Technical University of Athens, 15780 Athens, Greece; 2 Independent Geotechnical & Tunnel Engineering Consultant, N. Erythraia, 14671 Athens, Greece; gproun@gmail.com; 3 Engineering Geology & Geomechanical Monitoring Consultant, 18755 Athens, Greece; d_papouli@yahoo.gr; 4 Acropolis Restoration Service, Hellenic Ministry of Culture, 10555 Athens, Greece; ysma@culture.gr (D.M.); veleftheriou@culture.gr (V.E.)

**Keywords:** 3D geotechnical analysis, foundation stability, geotechnical monitoring, Agrippa monument, Athens acropolis, preservation of monument

## Abstract

This study focuses on the geotechnical evaluation of the foundation conditions of the Agrippa Monument at the Acropolis of Athens, aiming to propose interventions to improve stability and reduce associated risks. The assessment reveals highly uneven foundation conditions beneath the monument. A thorough collection of bibliographic references and geotechnical surveys was conducted, classifying geomaterials into engineering-geological units and evaluating critical parameters for geotechnical design. Geotechnical models were developed and 3D finite element analyses were performed. The qualitative evaluation of the foundation under static conditions indicates no immediate risk of failure, as no accelerated movement has been observed and the monument’s tilt remains well below critical values. Time-dependent settlements are not expected from any clay layers in the artificial fills. However, further soil compaction could occur due to seismic events, water action (causing erosion or voids), or changes in the monument’s weight or tilt under static conditions. The study also proposes instrumental monitoring, foundation soil improvement, and water management strategies to enhance the monument’s stability and mitigate potential risks.

## 1. Introduction

### 1.1. Preservation and Stability of the Agrippa Monument, General Context

The preservation of historical monuments is a critical intersection of cultural heritage and engineering science, particularly in the context of geotechnical uncertainties. The increasing attention to assessment, quantification, and mitigation of the risks affecting cultural heritage is reflected in the number of risk analysis methods developed for that purpose [1]. The significance and role of the geomechanics of structures in the problems faced worldwide with heritage and historic structures is underlined by the abundance of case studies where the risks affecting the structures’ integrity are related to their foundation conditions [2,3,4].

The Pedestal of the Agrippa Monument at the Acropolis of Athens serves as a prime example, as its foundation conditions present significant challenges that can jeopardize its structural integrity. This study seeks to conduct a comprehensive geotechnical evaluation of the monument’s foundation, with the aim of proposing effective interventions to enhance stability and mitigate associated risks.

Research in this domain increasingly highlights the complexity of assessing foundation conditions for historical structures, particularly given the inherent uncertainties in soil properties and behavior. Previous studies have shown that differential settlements and tilting, often arising from heterogeneous foundation soils, pose considerable risks [5]. For the Agrippa Monument, the foundation is uniquely challenged, resting partially on natural limestone bedrock and partially on artificial fills. The western side’s reliance on these fills and a pre-existing retaining wall that was incorporated into them complicates the stability dynamics, making it crucial to understand how these factors contribute to tilting and other structural failures.

Recent studies in the field have employed various approaches to assess the stability of historical structures, including finite element modeling and geotechnical risk assessment [6]. However, these studies often encounter controversy regarding the best methods to model uncertain soil properties and the effects of environmental changes, such as seismic activity and water infiltration. The present study seeks to address these divergent hypotheses by conducting a thorough analysis of the Agrippa Monument’s foundation conditions, classifying geomaterials into engineering-geological units and performing 3D finite element analyses.

By classifying geomaterials into distinct engineering-geological units and evaluating critical geotechnical parameters, a nuanced understanding of the monument’s foundation is approached. Our analyses reveal that, under current static conditions, there is no immediate risk of failure, as the observed tilt remains within safe limits. However, several key factors of uncertainty are identified, such as the variable properties of artificial fills, the performance of the classical ramp wall, and the historical context of previous structural reinforcements.

Ultimately, this work aims to enhance the stability of the Agrippa Monument through proposed interventions, including instrumental monitoring, soil improvement strategies, and effective water management. A combination of interventions is recommended, including instrumental monitoring to track the monument’s behavior over time, improvements in the foundation soil through grouting to enhance stability, and effective water management strategies to mitigate erosion and infiltration risks.

Hence, this study not only provides a thorough geotechnical evaluation of the Agrippa Monument’s foundation but also contributes to the broader understanding of managing geotechnical uncertainties in historical preservation. By focusing on both qualitative and quantitative assessments, the aim is to ensure the monument’s continued stability amidst the inherent challenges posed by its unique foundation conditions.

**Figure 1:**
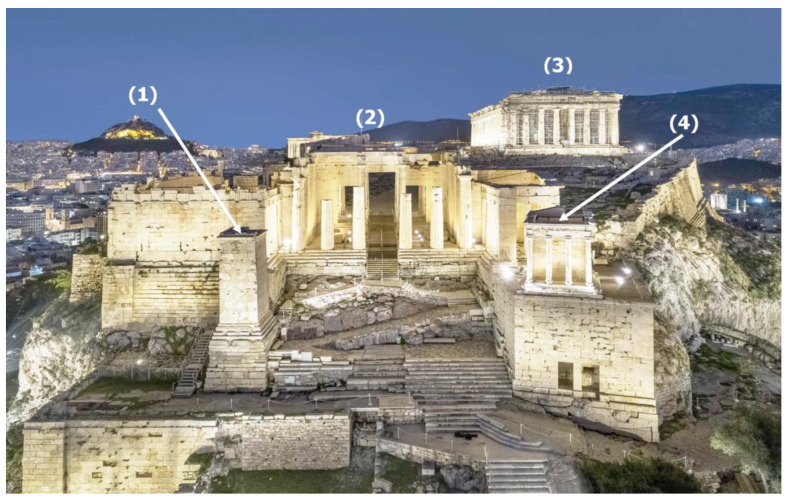
The position of the Agrippa Monument at the Propylaea at the famous Acropolis rock hill. (1) Agrippa monument, (2) Propylaia, (3) Parthenon, (4) Temple of Athena Nike (modified figure from website: https://www.onassis.org/el/news/ accessed on 6 November 2024).

The Pedestal of the Agrippa Monument is located to the west of the northwestern wing of the Propylaea at the famous Acropolis hill in Athens (Figure 1). Its construction has been attributed to the king of Pergamum Eumenes II (197–158 BC). At first, it supported a tethrippon (four-horse chariot) driven by himself and his brother Attalus II. During the 1st century AD, the tethrippon was replaced by a statue depicting the general and son-in-law of Emperor Octavian, Agrippa, as a charioteer.

**Figure 2:**
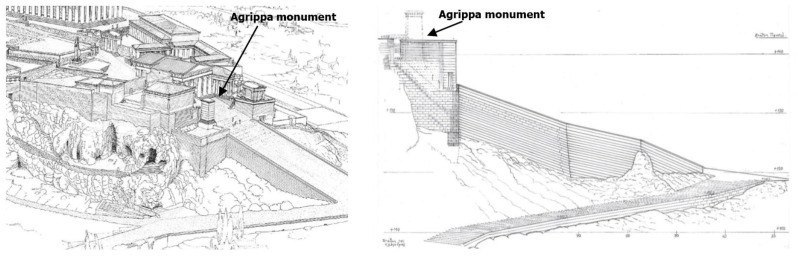
Graphic of the upper section of the Panathenaic Way and the northern façade of the ascent to the Acropolis, showing a cross wall in the foundation of the Agrippa monument [7].

The monument was erected on the existing deposit of the Mnesiclean revetment, almost parallel to the foundations of the north-western wing of the Propylaia, and was adjusted to the already built classical ramp leading to the Acropolis sanctuary (Figure 2). Part of the north retaining wall of this ramp was incorporated into the south half of the foundation of the Pedestal of Agrippa.

**Figure 3:**
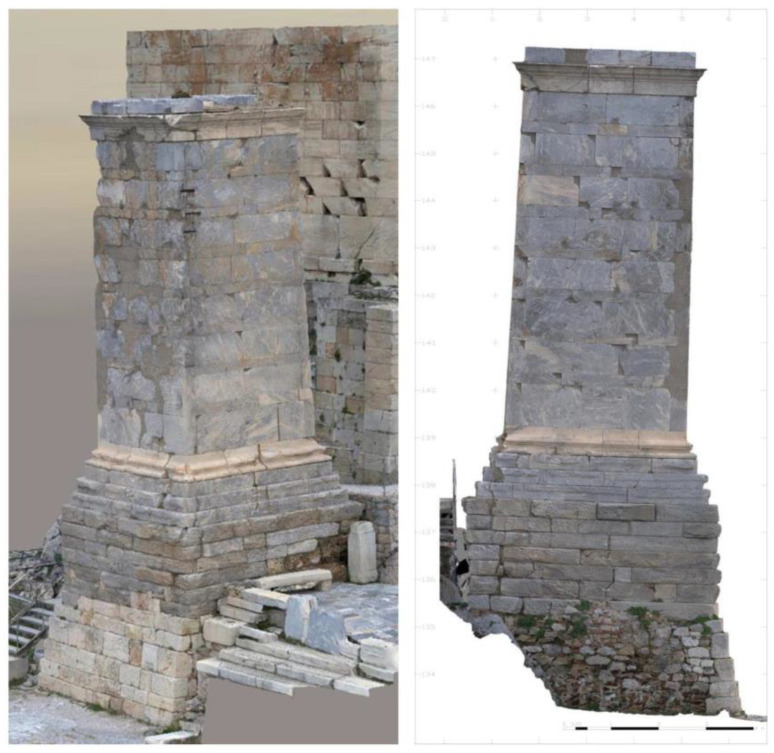
Photogrammetric recording of the southwestern (**left** figure) and north (**right** figure) section of the Agrippa Monument [8].

The Agrippa Monument consists of a base and a foundation, upon which the superstructure, the main shaft and the crown, rests (Figure 3). The foundation, the maximum height of which is 4.50 m, is made of Hymettus marble and conglomerate stones in the monument’s invisible parts. The lower part on the western side, about 2 m high, was repaired with porous stone in the late 19th century (Figure 3). On top of the foundation sits a three-stepped crepis, about 1 m high, made of Hymettus marble, an Attic-Ionic base about 0.6 m high, made from white Pentelic marble, a 7 m tall slightly tapering shaft built in the opus pseudo-isodomum system, built with Hymettus marble, and a 0.6 m high protruding crown of white Pentelic marble. The total height measured from the ground on the west side is approximately 13.7 m (Figure 3). The shaft has a rectangular section measuring 3.35 m by 3.9 m at the base, with a reduction of about 8% below the crown (dimensions 3.08 m by 3.6 m) (Acropolis Restoration Service, YSMA).

### 1.2. Historical Context

The Agrippa Monument has experienced tilting and structural failures primarily due to differential settlements from uneven foundation soils. Its foundation is situated on limestone bedrock in the eastern section, while the western part rests on artificial fills and a classical ramp retaining wall. Historically, several structural reinforcements have been implemented, including the addition of reinforced concrete, the construction of a retaining wall, and sealing of stone joints. Currently, ongoing reinforcement efforts focus on grouting and jointing in the foundation based on relevant structural studies.

**Figure 4:**
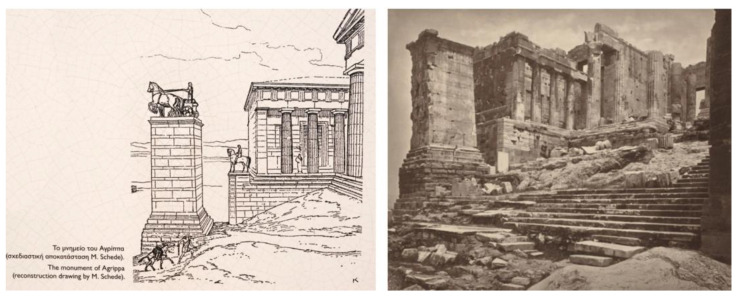
1869. The inclination and displacement of Agrippa’s base are noticeable. The artificial fill on the southern side of the base and the limestone rock mass in the eastern section can be seen (William James Stillman from ref [10] (**right** figure). 19 B.C. The Agrippa Monument. Restoration drawing by M. Schede. The rocky substrate in the eastern part of the monument is distinguished (from an information plaque at the monument) (**left** figure).

Old photographs reveal that tilting and displacement of the monument were evident as early as 1853 (Figure 4). Notably, in the 1880s, architect R. Bohn conducted excavations near the monument as part of his research, although significant work was limited due to concerns about the monument’s stability. Urgent conservation measures were necessary because of visible damage, including cracks and fragment detachments from centuries of degradation and sieges affecting the Acropolis [9].

Architect M. Korres noted the severe westward tilt of the monument, which, while not threatening total collapse, has led to significant structural issues. In 1865, repairs were made to the western foundation by reconstructing the lower section. A more extensive restoration occurred between 1914 and 1928, led by N. Balanos, that included repairs to the foundation and body of the monument. From the 1950s to the 1970s, continuous monitoring of the monument’s tilt was conducted using various techniques, including glass markers and crack meters. By the 1980s, sealing of cracks had also been implemented. Currently, the base of the Agrippa Monument shows a northwest tilt, with an approximately 25 cm displacement westward and 10 cm northward, indicating ongoing differential settling related to foundation behavior.

## 2. Materials and Methods

This study employs a multi-faceted approach to evaluate the geotechnical conditions of the Agrippa Monument’s foundation, integrating extensive literature reviews, geophysical investigations, and geotechnical surveys. Comprehensive field investigations included exploratory boreholes to determine the composition and characteristics of the underlying materials. Geophysical investigations, including seismic refraction and ground-penetrating radar (GPR), were utilized to identify the geological complexity of the foundation area, enhancing the understanding of subsurface conditions.

Laboratory tests were conducted to assess soil and rock strength and deformability parameters, which are essential for stability analyses. Laboratory tests included standard unconfined compressive strength (UCS) tests, point load tests, and classification tests (Atterberg limits, USCS, etc.) to evaluate the physical and mechanical properties of geomaterial samples. Additionally, video recording of the inner walls of the boreholes was carried out to identify the voids in the ground at the drilling locations.

Initial assessments classified the geomaterials into five distinct engineering-geological units, each characterized by unique properties crucial for geotechnical design. 2D and 3D engineering geological models were designed to support the 3D geotechnical analysis. To quantify the foundation’s performance under static and seismic loads, 3D finite element analyses were conducted using RS3 software from Rocscience. This involved modeling the monument’s superstructure, base, and the existing ancient wall alongside the surrounding geological context. Two groups of parametric analyses were executed: Group A focused on assessing the current deformational state to identify the mechanisms of observed tilting, while Group B aimed to simulate potential future deformations under different seismic scenarios.

The quantitative evaluation also included the application of grouting techniques to enhance the foundation soil’s properties, while water management strategies were integrated into the methodology, focusing on sealing gaps at the monument’s base to prevent water infiltration.

## 3. Geological Complexity Below the Historical Structure

### 3.1. Geological Conditions

**Figure 5:**
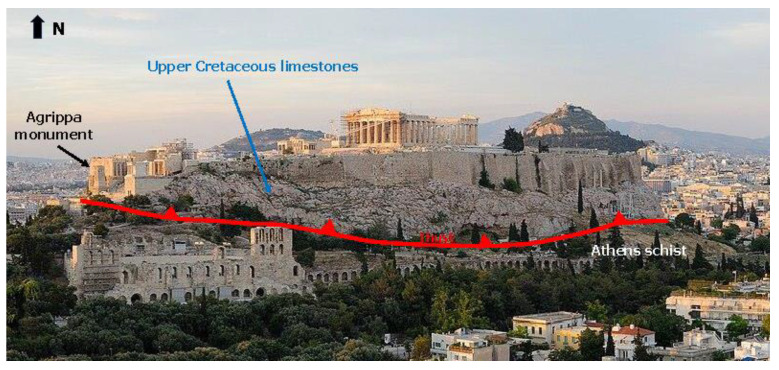
The thrust of the Upper Cretaceous limestone on the Athens schist on the Acropolis hill (modified figure from website: https://www.ellines.com/en/ accessed on 6 November 2024).

The geological environment of the Acropolis hill in Athens has been the subject of study by various researchers since the mid-18th century, including notable studies by [11,12,13,14,15], among others. At the base of the hill, the Athenian schist is present, while the summit is characterized by an allochthonous sequence of Upper Cretaceous limestones (Figure 5). The limestones of the allochthonous sequence are thick-bedded to unbedded, primarily inclined towards the interior of the hill [16]. In some areas, they are crystalline and clastic. They often appear intensely fractured. There is significant development of karst features, particularly on the northern and eastern sides of the hill, with large dimensions. The maximum thickness of the allochthonous limestones is approximately 40 m.

Interspersed between these formations is tectonic agglomerate, a product of the thrusting of limestones over the Athens schist [17,18]. The tectonic agglomerate primarily consists of limestone and, in some areas, schistose sandstone agglomerates. Locally, large lenses of compact grey limestone or layers of fine-grained red limestone and less frequently siliceous rocks are encountered. The matrix material is primarily calcareous marl and clayey sandstone. This horizon sometimes exhibits layering. The thickness of the formation varies along the southern slope, reaching zero in some areas. The maximum thickness is found in the western part of the hill, where it exceeds 10 m [19].

A key characteristic of the Acropolis hill’s tectonics is the compression and consistent structural alignment of the geological formations [20,21,22]. The limestones, agglomerates, and Athens schist are oriented along a folding axis that runs in an east-west direction. Typically, these layers incline towards the interior of the hill, exhibiting a convergent structure with a gentle slope. The limestone shows large-radius folding, while the Athens schist displays a folded or microfolded structure [23]. Furthermore, the tectonic landscape is marked by faults, particularly concentrated around the northern section of the hill, leading to displacements of limestone masses relative to the underlying formations. The main fracture directions in the limestone layers align with the East-West axis, prominently visible on the northern and southern flanks, while an additional younger north-south fracture system is also present.

### 3.2. Engineering Geological Evaluation

The geological and engineering-geological survey of the Agrippa Monument’s foundation area uncovered considerable geological complexity and notable differences in the subsurface geomaterials. The eastern portion of the monument, facing the Propylaea Gallery, rests on Upper Cretaceous limestones, while the western section is founded on artificial fill materials, highlighting the variation in foundational support across the monument.

**Figure 6:**
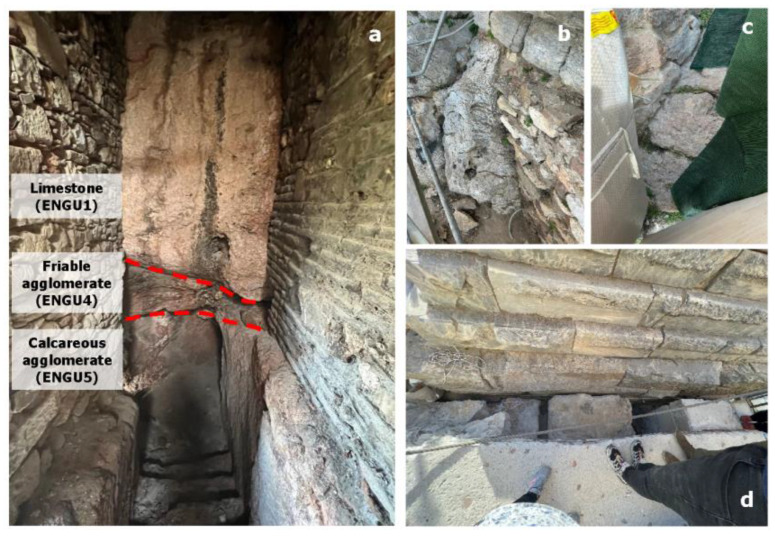
(**a**) Stratigraphy of geoformations in the underground space north of the monument within the artificial embankment toward the northern walls. (**b**) The limestone at the northeast corner of the monument’s foundation. (**c**) The limestone at the southeast corner of the monument’s foundation. (**d**) The ancient wall, 1.5 to 2 m wide, located beneath the foundation in the eastern section.

In the eastern section, thick-bedded to massive limestone, which is slightly to moderately fragmented, was observed. In some locations, crystalline and karstified limestone is present. The limestone exhibits a blocky structure that interlocks well and, where visible, the layers incline toward the interior of the hill. On the northeastern side of the monument, a steep incline of the limestone surface toward the west (fault: 64/260) is evident (Figure 6b), indicative of the tectonic activities around the hill and the effects of faults, which cause displacements of limestone masses relative to the underlying geological formations.

The underlying limestone formation, according to the geotechnical investigation program [24,25,26,27] and mapping of an underground space, located north of the monument within the artificial embankment, consists of a tectonic agglomerate (Figure 6). The contact between the limestone and the underlying agglomerate is nearly horizontal, with both limestone and conglomerate layers aligning in a concordant structure. At the interface between the limestones and the agglomerate, a zone of poorer quality agglomerate was identified. This agglomerate is friable, highly weathered, and intensely fragmented, containing limestone-marl and clayey-sandy binding material. The underlying agglomerate of this poorer quality zone is calcareous and only slightly to moderately fragmented, with a blocky structure with very good interlocking.

The western part of the monument is founded on artificial deposits, which mainly consist of very loose to loose clayey gravels, with mixed conditions of sand, gravel, clay, and silt. Fragments of ancient masonry stones are also encountered in some areas.

During the conservation work on the eastern section of the monument, it was found that the monument is partially founded on a transverse ancient wall (classical ramp) oriented in the east-west direction, with a width of approximately 2 m (Figure 6d).

**Figure 7:**
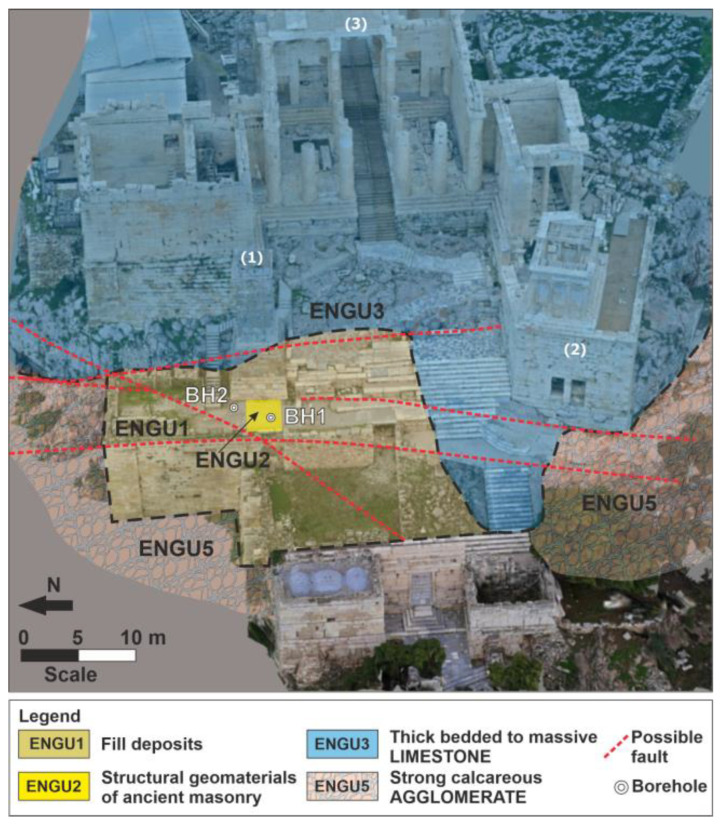
Engineering geological map—2D engineering geological model of the Agrippa monument area. (1) Agrippa monument, (2) Temple of Athena Nike, (3) Propylaia.

**Figure 8:**
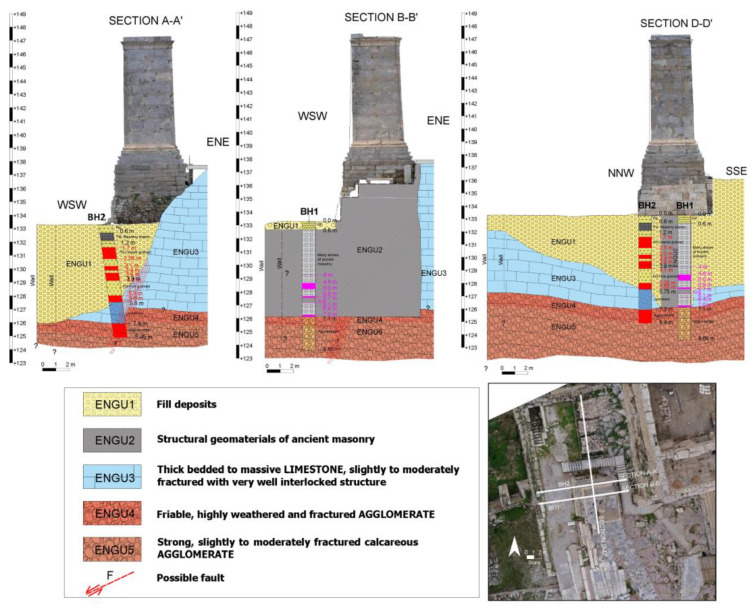
Engineering geological cross sections—2D engineering geological model of the Agrippa monument area.

**Figure 9:**
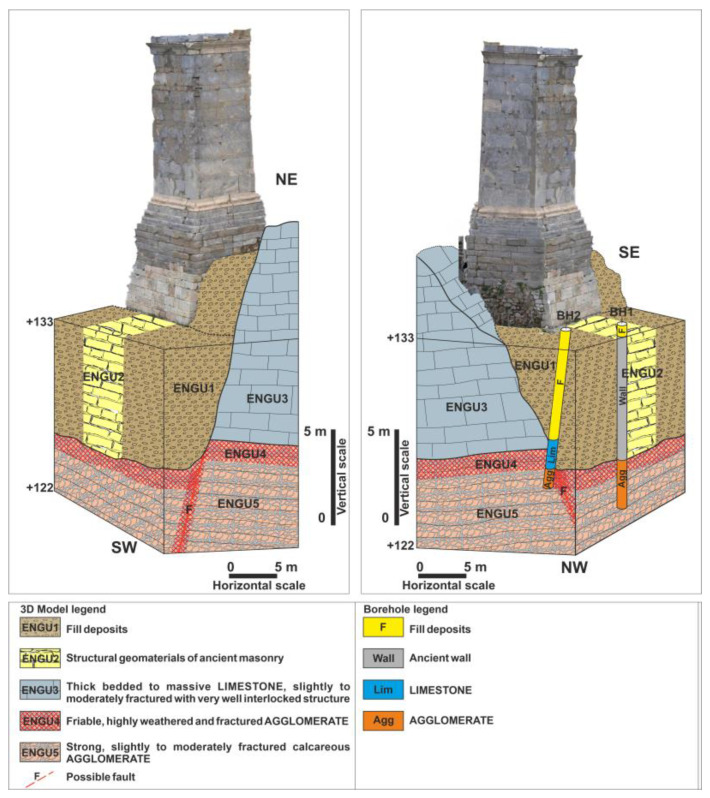
3D engineering geological model of the Agrippa monument area.

To investigate the stratigraphy and engineering-geological characteristics of the fills deposits and rocky formations at the monument’s foundation, two sampling boreholes were drilled (BH1, a vertical borehole reaching a depth of 9.5 m, and BH2, an inclined borehole angled at 8° towards the east, approximately 8.5 m deep) (Figure 7, Figure 8 and Figure 9) [27]. Video recordings were made inside these boreholes to identify voids, while laboratory tests determined the mechanical properties of the geological materials and geophysical surveys were conducted.

The fill deposits exhibit significant heterogeneity in both nature and quality, with numerous voids present. The rocky bedrock of the Acropolis hill was found at depths of 6 to 7 m in the boreholes, consisting of Upper Cretaceous limestone and the overlying agglomerate.

The geophysical methods employed included Ground Penetrating Radar (GPR) and, as a complementary approach, electrical tomography. The tomographies revealed low-resistance zones near the surface and at a depth of 2 m, located west of the monument’s foundation (near the northwest and southwest corners), indicating moisture accumulation from water flow. Additionally, a residual wall, approximately 1.5 to 2 m wide, was identified on the western side of the foundation, represented by a high-resistance zone near the northwest corner.

**Figure 10:**
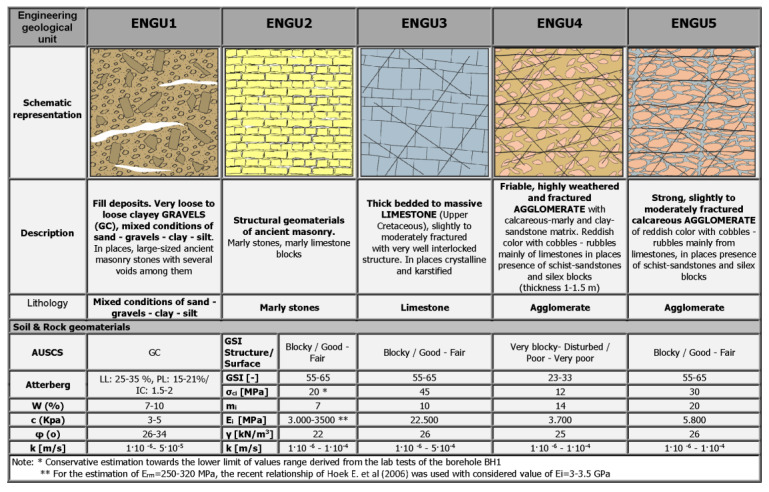
Geotechnical design parameters for each engineering-geological unit [28].

During the geological and engineering-geological mapping, GSI rock mass classifications were carried out on the rocky formations (Figure 10) and discontinuity measurements were recorded using a geological compass.

Considering all the aforementioned factors, including the geological structure and origin, lithological composition, primary (inherent) structure (such as stratification), secondary (acquired) structure (including folding, fracturing, and disturbance), the quality of discontinuities, and the expected behavior alongside potential failure mechanisms in a foundation environment, the geomaterials in the monument area were categorized into five engineering-geological units, each characterized by unique properties crucial for geotechnical design (Figure 10).

- ENGU1: Fill deposits. Very loose to loose clayey Gravel (GC), mixed conditions of sand—gravels—clay—silt. In places, large-sized ancient masonry stones with several voids among them.
- ENGU2: Structural geomaterials of ancient masonry. Marly stones, marly limestone blocks.
- ENGU3: Thick bedded to massive Limestone (Upper Cretaceous), slightly to moderately fractured with very well interlocked structure. In places crystalline and karstified.
- ENGU4: Friable, highly weathered, and fractured Agglomerate with calcareous-marly and clay-sandstone matrix. Reddish color with cobbles—rubbles mainly of limestones in places presence of schist-sandstones and silex blocks (thickness 1–1.5 m).
- ENGU5: Strong, slightly to moderately fractured calcareous Agglomerate of reddish color with cobbles—rubbles mainly from limestones, in places presence of schist-sandstones and silex blocks.

The engineering-geological conditions at the monument area are characterized by significant heterogeneity in the foundation. The eastern side of the monument is found on limestone bedrock (ENGU3), whereas the western side is situated on fill deposits (ENGU1), reaching a maximum depth of approximately 7 m. The fill displays considerable variability in both material nature and quality, including large ancient masonry stones and numerous voids. Furthermore, the monument is partially situated on a transverse ancient wall composed mainly of weak marly stones (ENGU2), oriented in an east-west direction and ~2 m wide. A steep fault, inclined at about 70° to the west, displaces the rock formations of ENGU3, ENGU4, and ENGU5. ENGU4 is predominantly found in the contact zone between ENGU3 and ENGU5, as well as along the fault. The contact between ENGU3, ENGU4, and ENGU5 is nearly horizontal, with a slight slope toward the east into the interior of the hill (Figure 7, Figure 8 and Figure 9).

Engineering geological mapping of the Agrippa monument area was carried out (Figure 7).

2D and 3D engineering geological models (Figure 7, Figure 8 and Figure 9) were designed to support the 3D geotechnical analysis. Also, engineering geological cross sections were compiled (Figure 8).

The geotechnical design parameters for each engineering-geological unit in the foundation area of the Agrippa monument, detailed shown in the following table (Figure 10), are based on a synthesis of laboratory test results from the geotechnical investigation, the range of design parameters for the geological formations of the engineering-geological units derived from related projects in the broader study area, and the experience of the research team.

### 3.3. Hydrogeological Conditions

**Figure 11:**
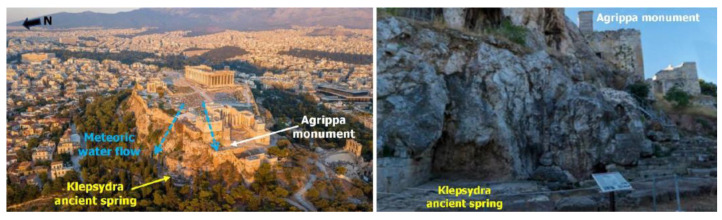
Meteoric water flow, influenced by the topography of the Acropolis hill, directs northwest toward the Agrippa monument and the Klepsydra spring (**left** figure). The location of the Agrippa monument is visible at the Klepsydra spring (**right** figure) (modified figure from website: https://exploringgreece.tv/athina/ accessed on 6 November 2024).

The contact between the Athenian schist and the overlying limestone creates an ideal environment for karst sinkholes and an underground aquifer, primarily due to the morphology of the interface, which is shaped by folds. The karstic and fractured limestone serves as an aquifer, unlike the generally impermeable Athens schist. This underground morphology allows aquifer discharge through springs like Klepsydra (Figure 11), located near the northwest section of the Agrippa monument, as well as smaller springs on the southern side of the Acropolis hill [29,30].

Meteoric waters flow from the southern and eastern sides of the hill to the northwest, particularly towards the Agrippa monument and Klepsydra spring (Figure 11), exacerbating erosion of the underlying rocky slope and the artificial plateau supporting the Agrippa monument [31,32]. This runoff leads to internal erosion and further loosening of the fill materials beneath the monument.

## 4. Geotechnical Evaluation of Foundation Conditions

The present section evaluates the geotechnical conditions of the monument’s foundation, aiming to:

- Summarize the critical factors and parameters affecting the response of the foundation area of the monument with references, when required, to the structure itself.
- Evaluate the existing geotechnical risk associated with the correct estimation of the representative values of the strength and deformability parameters of geomaterials and structures, which have an intrinsic uncertainty.
- Perform a qualitative assessment of the degree of safety of the monument’s foundation, based on the available data and the basic soil mechanics principles.
- Perform a quantitative assessment of the degree of safety and the deformational state of the monument’s foundation via 3D finite element numerical analyses.

### 4.1. Geotechnical Risk and Uncertainties

The source of the deformation and tilting of the Agrippa monument is obviously related to its foundation. Specifically, the deformation observed is due to the intensively heterogeneous foundation conditions, as the monument is founded on the competent limestone rock mass on the east side and on artificial deposits on the west side. On the west side, the monument is also partially founded on the existing ancient masonry wall, which is approx. 2.2 m thick, with an east-west direction, which is found slightly to the south of the monument’s axis. This eccentric position of the wall may be the cause of the slight tilting of the monument to the north, while the main tilting direction is to the west.

Predicting/modelling the actual deformation of the monument is, however, not a simple task, as this deformation is a result of combined effect of the following critical factors:

1. The geotechnical characteristics of the artificial deposits, which exhibit:
  - Intense heterogeneity and poor grading, with frequent alternation of (mainly) coarse-grained and (secondarily) fine-grained soil.
  - Incomplete compaction and potential existence of voids that, due to the limited foundation area of the monument, could greatly impact the temporal and future response of the monument, even if appearing locally and in isolation.
  - Sensitivity to possible water movement that can cause washing out of fine-grained soil and piping, followed by further settlement under constant loading.

Due to the above, the estimation of representative parameters for the artificial deposits involved significant uncertainties, which are aggravated by the limited dimensions of the foundation that render even local heterogeneity or voids significant and reduce the reliability of representative parameters based on statistical processes over large amounts of relevant data.

2. The strength and deformability characteristics of the existing ancient masonry wall, as well as the seating conditions of the monument on it. The wall has been built on the bedrock. The investigation revealed that the wall consists of stones of various origins (porolith, marl, marly limestone) and strengths, with defects and detachments, which reveal that the wall has been considerably damaged. Hence, estimating the strength and deformability parameters of the wall, as well as the conditions (in the sense of smoothness, flatness, and condition of the upper surface of the wall) of the seat of the monument on it, also involves high levels of uncertainty.
3. The strength and deformability characteristics of the base and the foundation of the monument. Unlike the superstructure of the monument that seems to have rotated as a solid body, the foundation and (mainly) the base of the foundation, consisting of stonewalling with stones of shale and conglomerate origin, have exhibited intense inelastic response with serious pathogeny and cracking. It is hence rationally considered that the current tilting/deformation of the monument is not only a result of differential settlement of the base, but also of the deformation of the base and the foundation themselves due to intense plasticization.

As there has been no possibility of monitoring the settlement of the monument’s foundation and as the only observation refers to the tilting angle, it is not known which part of the tilting is due to settlement and which is due to inelastic deformation of the foundation and the base (opening of cracks, dilation of joints).

Additionally, the current state does not suggest a profound failure in the sense of a collapse or a deep failure of the foundation, but a serviceability failure due to differential settlements. In such cases, back analyses have limited value in the reduction of geotechnical uncertainties, as there are multiple combinations of geotechnical parameters that could result in the same deformational response of the monument. Every such combination produces a different stability factor for the monument in static and (mainly) seismic conditions. Therefore, it has been decided to approach the evaluation of the stability conditions of the monument regarding its foundation through parametric analyses of the strength and deformability of the in situ and man-made materials involved, reaching conclusions that are more probabilistic than deterministic.

### 4.2. Qualitative Assessment of Foundation Conditions Under Static Loads

The current condition of the monument does not indicate an immediate hazard for foundation failure. Although there is no detailed record of its deformational response over time, it is obvious that during the last decades, there has been no accelerated movement that indicates a tendency to collapse, whereas the current tilting is far below the value required to cause toppling.

Additionally, due to the nature of the artificial deposits (clayey gravels to locally clayey sand), as these were retrieved from the respective exploratory boreholes, no considerable time-dependent settlements (consolidation/creep) are expected. Such settlements would be expected if the foundation of the monument was on fine soils of considerable spread and depth. Therefore, any settlements are expected to be completed within a short period of a few months after any considerable change in the stress field. This means that, since the load on the ground due to the monument and the retaining structures around it remain the same for several decades, any compression of the soil in its foundation is complete.

Additional settlement/compression of the soil can only happen due to:

- Seismic event. During the lifetime of the monument, many earthquakes have taken place. If no earthquake considerably stronger than the historic earthquakes takes place, no considerable additional consolidation of soil below the foundation is expected, which could cause additional tilting of the monument. However, if a seismic event caused additional tilting of the monument due to permanent damage of its base and foundation, the resulting locally increased stresses on the ground would cause a corresponding settlement.
- Action of water. Ingress and circulation of water within the deposits and around the existing ancient wall could cause washing out of soil and voids/piping. Such voids, if created, are then compressed under the load of the monument, causing additional settlements.
- Increase of weight or additional tilting of monument under static conditions. In case of (a) significant increase of the monument’s weight due to maintenance/restoration works or (b) further opening of cracks and tilting due to the time-dependent behavior of the mortar between the rock blocks, the stress found in the foundation will change, resulting in further differential settlement.

Summarizing the above, the foundation of the monument under static conditions is not expected to suggest a significant risk for collapse, given that:

1. Infiltration and circulation of water in the foundation area is minimized.
2. The base and foundation of the monument are repaired so that the structural deficiencies are rectified.

### 4.3. Quantitative Assessment of Foundation Conditions

For the quantification of the geotechnical risk related to the foundation of the Agrippa monument, the use of simplified analytical methodologies was rejected, as:

The foundation conditions are intensely heterogeneous, particularly across the E-W and the N-S axis.

The performance of the foundation is not evaluated in terms of an ultimate limit state criterion, but with the aim to avoid additional differential settlement, especially under seismic loads, so as not to cause additional stress concentration, deformation, and tilting of the monument.

There is increased uncertainty related to the distribution of stresses due to the weight of the superstructure and due to the different materials encountered below its foundation level. This distribution also depends on the properties of the monuments base and foundation structure.

For the above reasons, the evaluation of the foundation conditions was performed using 3D, finite element numerical analyses. Software RS3 from Rocscience (version 4.034) was used.

#### 4.3.1. Presentation and Assumptions of Executed Analyses

In the 3D, finite element models used for the present study, the monument and the surrounding structures and geomaterials (man-made and in situ) were modelled using 4-Noded Tetrahedra in a graded (not uniform) mesh. No structural elements (beams, plates, or shells) were used due to the nature of the structures (masonry) and the main scope of the study (response of the foundation to the applied static and dynamic loads). The dimensions of the model were kept to a minimum (length x = 40 m, width y = 25 m, and depth z = 15 m below the monument foundation) to reduce the calculation time. These dimensions did not affect the results of the analyses due to the limited dimensions of the monument’s foundation (approx. 6 m × 6 m) and the very high strength and stiffness of the bedrock.

**Figure 12:**
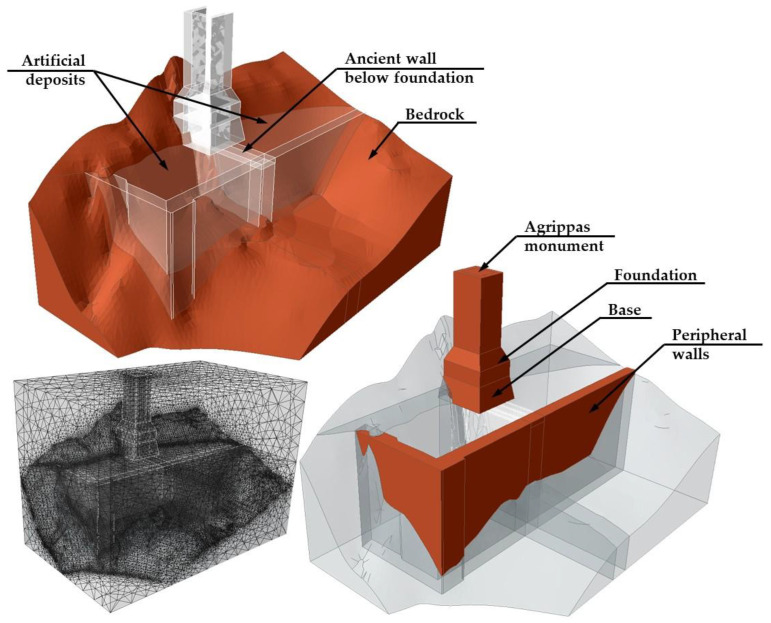
Aspects of the finite element model used.

The figure below (Figure 12) illustrates indicative aspects from the finite element models used in the analyses conducted.

The cases analyzed per material/structure in the parametric analysis performed are summarized in the following points:

1. Monument superstructure (structure above the foundation). Due to its observed tilting as a solid body without obvious defects, the superstructure was modelled as a homogeneous, elastic material, with indicative elasticity modulus equal to 1 GPa.
2. Monument base and foundation. These parts of the structure were parametrically considered with both elastic and elastic—perfectly plastic response, following Mohr—Coulomb failure criterion, using σ_c_ = 1.00 MPa and σ_t_ = 0.010 MPa, respectively. The elasticity modulus was conservatively considered at E_base_ = 300 MPa. The response after the restoration (grouting) works was also considered, increasing the strength to σ_c_ = 2.00 MPa and σ_t_ = 0.020 MPa, respectively, and the elasticity modulus to 1.5 GPa.
3. Existing ancient masonry wall at the foundation. The buried ancient wall was modeled as an elastic—perfectly plastic material, following the Hoek–Brown failure criterion, using the parameters shown in the table below (Table 1). The failure criterion was selected as a solution to be implemented in the specific software and not as the best solution for such material. To avoid a very wide range of analysis, the strength was selected using specific conservative set of parameters, whereas the deformation modulus was examined parametrically.
4. Peripheral retaining walls. Elastic response was considered as the walls appear to have no defects and their effect on the foundation conditions of the monument is considered minor. The walls were modelled as a homogeneous, elastic material, with indicative elasticity modulus equal to 1 GPa.
5. Artificial deposits. The deposits were modelled as elastic—perfectly plastic materials following the Mohr–Coulomb failure criterion. Their properties were parametrically examined within the range shown in the table below (Table 2).
6. Bedrock. The bedrock was modeled as an elastic—perfectly plastic material, following the Hoek–Brown failure criterion. The characteristics of the bedrock are such that they are not considered a critical parameter of the problem.

Two groups of analyses were conducted:

- Analyses’ group A. In this group, a parametric analysis of the aforementioned cases and parameters was performed, so as to approach its current deformational state (tilting). The scope of this group was to (a) confirm the mechanism of development of today’s tilting of the superstructure and (b) limit the range of parameters in view of the next group of analyses. The analyses considered structural loads (construction of the monument) and seismic loads (pseudo-static application of a frequent earthquake—ag = 0.096 g, TR = 100 years) on the foundation. The analysis stages were the following:
  a. Stage 1. Geostatic conditions with consideration of only the bedrock.
  b. Stage 2. Activation of perimeter retaining walls and buried wall at the foundation.
  c. Stage 3. Activation of artificial deposits.
  d. Stage 4. Construction/activation of the Agrippa monument (application of static load on the foundation).
  e. Stage 5. Application of frequent earthquakes (ag = 0.096 g, TR = 100 years).

- Analyses’ group B. In this group, the current deformational state of the monument was modelled from the beginning (initial geometry) to predict the effect of a frequent and a rare (ag = 0.16 g, TR = 475 years) seismic event on the differential settlement and the tilting of the monument. The base and the foundation of the monument were modelled with the improved parameters corresponding to the restoration (grouting) works executed at the time of preparation of this study. The analysis stages were the following:
  a. Stage 1. Geostatic conditions with consideration of only the bedrock.
  b. Stage 2. Activation of perimeter retaining walls and buried wall at the foundation.
  c. Stage 3. Activation of artificial deposits.
  d. Stage 4. Construction/activation of the Agrippa monument (application of static load on the foundation), with its current state of deformation incorporated in the mesh.
  e. Stage 5. Application of frequent earthquakes (ag = 0.096 g, TR = 100 years).
  f. Stage 6. Application of rare earthquakes (ag = 0.16 g, TR = 475 years).

#### 4.3.2. Results and Discussion

**Figure 13:**
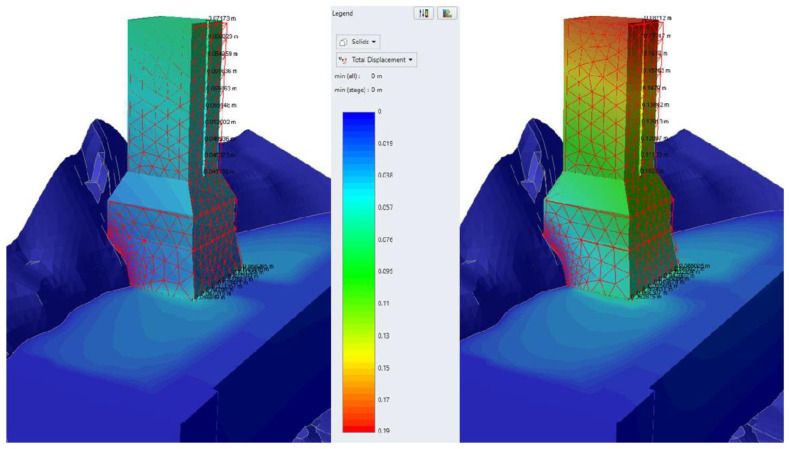
Illustration of total displacements results for static (**left**) and seismic (**right**) conditions for Analysis A8.

The main results of characteristic analyses of group A are summarized in the following table (Table 3). Representative graphic results are illustrated in Figure 13.

The basic conclusions from group A analyses are the following:

- To approach the observed tilting of the monument, an elastoplastic response of the base and the foundation must be assumed. Assuming an elastic base and foundation does not result in the observed tilting, even when considering the most conservative parameters for the deposits and omitting the buried wall.
- The theoretically calculated tilting approaches that observed on the monument when assuming (a) the lowest bound of the parameters for the deposits and absence of the buried wall or (b) conservative parameters for the deposits and reduction of the strength of the base and foundation of the monument after initial failure.
- The mechanism of the present deformation of the monument depends on many parameters that cannot be accurately quantified due to important existing uncertainties. The most probable geotechnical models of the foundation conditions are those of analyses A6 and A8. The additional effects of the following factors may have shaped the current deformational condition:
  a. The actual conditions (planarity, defects, geometry) of the foundation of the monument on the ancient, buried wall, which cannot be known or properly modelled.
  b. Local/intense heterogeneity (existence of voids or boulders) within the artificial deposits, which may cause differential response of the deposits themselves (on the north and south side of the vertical, buried wall).
  c. Cumulative effect of multiple seismic events with various orientations and magnitudes causing incremental damage to the monument and residual plastic deformations, which also cannot be modelled properly.

Summarizing the above, it is considered much more important to perform actions that limit the uncertainties related to the foundation conditions and their obvious heterogeneity than to attempt to accurately model these conditions.

**Figure 14:**
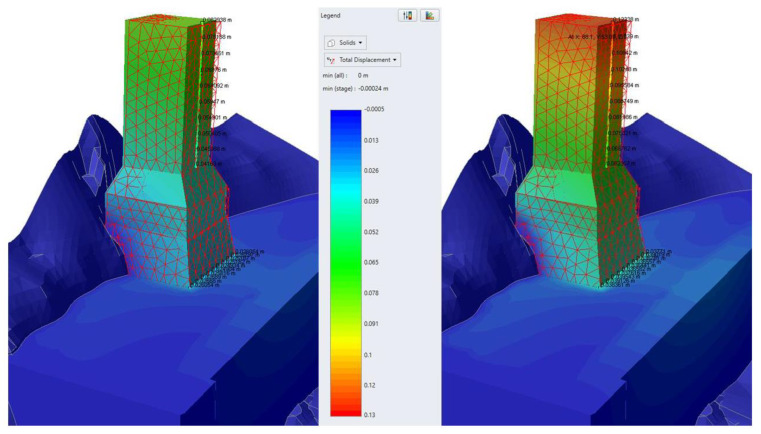
Illustration of total displacements results for frequent (**left**) and rare (**right**) seismic events for Analysis B2.

**Figure 15:**
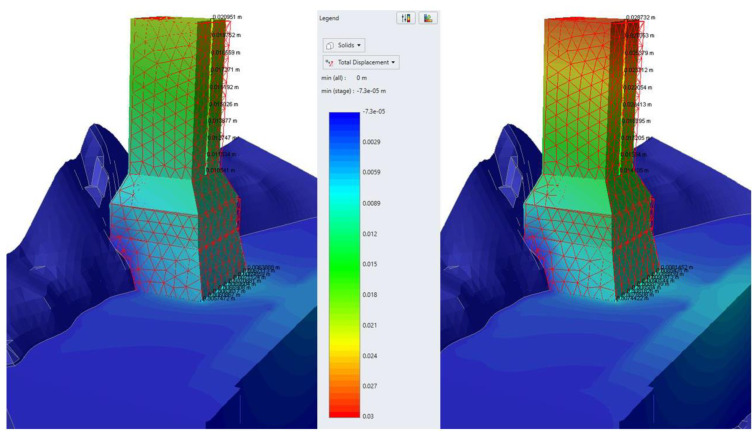
Illustration of total displacements results for frequent (**left**) and rare (**right**) seismic events for Analysis B5.

**Figure 16:**
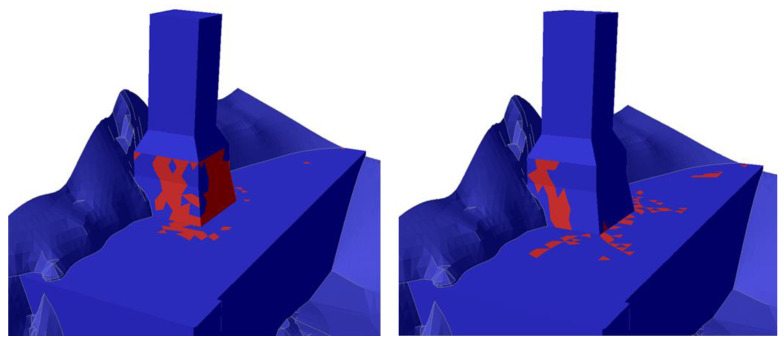
Illustration of plastic elements (red area) for rare seismic events from analysis B2 (**left**) and analysis B5 (**right**).

The main results of characteristic analyses of group B are summarized in the following table (Table 4). Representative graphic results are illustrated in Figure 14, Figure 15 and Figure 16.

The basic conclusions from group Β analyses are the following:

- Considering elastic response for the base and the foundation of the monument (analysis B1), the theoretically calculated additional settlement of the monument for frequent and rare seismic events is minor. The corresponding horizontal displacement at the crest of the monument does not exceed 13 mm.
- However, assuming elastic—perfectly plastic response for the base and the foundation of the monument (analyses B2–B3), the theoretically calculated additional settlement of the monument for frequent and rare seismic events is very considerable, exceeding 30 mm for a rare seismic event. The corresponding horizontal displacement at the crest of the monument approaches 6–8 cm for frequent and 10–12 cm for rare earthquakes.
- Considering the results of analyses B2–B3 not acceptable, the effect of consolidation grouting in the artificial deposits was investigated. Specifically, the parameters of the deposits were improved to c = 50 kPa, φ = 32°, and E = 80 MPa, which are feasible because of systematic grouting in the foundation area of the monument.
- Considering improved parameters for the deposits and elastic response for the base and the foundation of the monument (analysis B4), the theoretically calculated additional settlement of the monument for frequent and rare seismic events is minor. The corresponding horizontal displacement at the crest of the monument does not exceed 11 mm.
- Assuming improved parameters for the deposits and elastic—perfectly plastic response for the base and the foundation of the monument (analyses B5–B6), the theoretically calculated additional settlement of the monument for frequent and rare seismic events is very limited (≤8 mm). The corresponding horizontal displacement at the crest of the monument is controlled to values up to 27 mm for rare earthquakes (>75% reduction compared to analysis B2).

In summary, the application of consolidation through grouting in the foundation area of the monument, through the achieved improvement and homogenization of the properties of the deposits, minimizes the existing uncertainties and drastically reduces the theoretically anticipated additional deformation, even in the scenario of a rare earthquake. This result is indeed not strongly dependent on the response of the base and the foundation of the monument, which is also important.

## 5. Discussion

As derived from the geological and geotechnical evaluation of the immediate area around the monument, highly uneven foundation conditions are encountered, since this is founded on the eastern side on moderately fractured but blocky limestone bedrock and on the western side on artificial backfill materials, of maximum depth of approximately 7 m. In addition, the monument is partially founded on a wall (classical ramp wall) oriented east-west.

Regarding the geotechnical risks, the critical factors affecting the deformation of the monument are as follows:

Geotechnical characteristics of artificial fills:

- Heterogeneity
- Incomplete compaction
- Water circulation

Characteristics of the transverse classical ramp wall and the conditions of the monument’s foundation on it:

- Composition of stones, fractures—damages
- Smoothness, flatness, condition of the bearing surface
- Characteristics of the monument base and foundation:
- Significant inelastic behavior
- Contribution to the tilting of the monument

Groundwater within the limestone discharges through springs, such as the Klepsydra (northwest section near the Agrippa monument) and smaller springs on the southern side of the Acropolis hill, and does not pass into the foundation soil. On the other hand, meteoric water, due to the hill’s geomorphology, flows from the southern and eastern sides to the northwestern part, towards the Propylaea area and the Klepsydra spring, eroding the underlying rocky slope in the broader cave area and the artificial foundation plateau of the Agrippa monument. Water infiltration into the backfill materials causes internal erosion and further loosening of the artificial fills on which the monument rests.

From the qualitative evaluation of the foundation conditions under static conditions, no immediate risk of foundation failure is observed, as no accelerated movement has been detected and the tilt is significantly smaller than critical values. It is also noted that no significant time-dependent settlements are expected from any clay layer that may exist in the artificial fills of the monument’s foundation.

Further compaction of the soil under static conditions may occur only due to: (i) a seismic event, (ii) water action (soil washing and void creation), (iii) changes in the weight or tilt of the monument under static conditions.

Thus, the condition of the monument under static conditions is considered stable, provided that:

1. Water infiltration and circulation in the foundation area of the monument are controlled.
2. The pathology of the monument’s superstructure (base and foundation) is improved. Relevant works have been proposed by the recent structural study [8], which are in progress at the time of this report’s completion and are expected to significantly enhance the resilience of the monument ‘s superstructure.

The key conclusions from the geotechnical evaluation using 3D numerical analyses with finite element calculations are:

- Considering an elastic–perfectly plastic behavior of the monument’s foundation and base (most rational assumption), the theoretically calculated additional settlement on the west side of the monument, in the case of frequent and rare earthquakes, is significant. The corresponding additional horizontal displacement at the top of the monument may reach 8 cm for a frequent earthquake and 12 cm for a rare earthquake.
- Judging that this result is unacceptable, improved parameters were considered for the artificial fills, which are deemed achievable through systematic consolidation grouting.
- With improved backfill properties, the theoretically calculated additional settlement on the west side of the monument during a frequent and rare earthquake is limited to ≤8 mm. The corresponding additional horizontal displacement at the top of the monument is significantly reduced to 27 mm (4.5 times smaller) for the rare earthquake.
- It is clearly more important to take actions that reduce the uncertainty of the foundation conditions and limit their evident heterogeneity, rather than attempting a more precise simulation of these conditions.

Based on the above, the actions that have been proposed regarding risks related to the foundation area of the Monument of Agrippa are improvement/strengthening of the foundation soil behavior below the monument through grouting and prevention of in-filtration into the monument foundation area.

### 5.1. Improvement of Foundation Soil Behavior Through Grouting

Based on the quantitative assessment of the expected deformational behavior of the monument, it has been proposed to improve the artificial backfill materials in the monument’s bearing area using cement grouting. The design, execution, and confirmation of the effectiveness of the grouting works shall be undertaken by a specialized contractor with proven experience in similar works.

The area recommended for improvement through grouting, in addition to the foundation soil under the monument, should extend at least 3.5 to 4 m around the monument on its northern, southern, and western sides. The depth of improvement should be at least 5 m. These minimum dimensions are naturally limited locally by the rocky substrate and the perimeter wall to the west. The minimum parameters that must be achieved for the improved soil are:

- Cohesion c = 50 kPa,
- Friction angle 32°,
- Modulus of deformability E = 80 MPa.

Grouting will be conducted without pressure (by gravity) through small-diameter boreholes of indicative depth 4–5 m, performed manually or with very light mechanical equipment, based on existing access restrictions and the contractor’s available equipment.

A suitable grid of vertical and inclined boreholes will be designed and applied around and beneath the monument. The grouting will be executed in appropriate (different) phases (primary, secondary, etc.). Systematic absorption measurements will be taken during primary, secondary, etc., grouting phases, so that the completion of each phase is determined based on pre-established performance criteria.

The achievement of the minimum strength and deformability values for the improved soil must be checked and confirmed by taking samples of the improved soil and conducting the corresponding tests of mechanical properties.

The execution of the soil improvement works will be carried out strictly and only after the installation of the proposed monitoring instrumentation systems.

### 5.2. Water Control—Prevention of Infiltration into the Monument Foundation

In addition to the recommendations regarding the broader management and regulation of surface water, which, due to the geomorphology of the Acropolis hill, flows from its southern and eastern sides to its northwestern part, it is also deemed necessary to take measures to prevent water infiltration in the immediate area of the monument, mainly on its eastern side. During the preparation of this report, significant gaps were observed at the foundation level of the monument, which clearly allow water to flow beneath the base of the monument into its foundation soil.

It has therefore been proposed to seal these gaps using non-shrinking grout. The grout should be applied in such a way that the final surfaces direct local rainfall away from the monument’s base and toward the nearest drainage system.

### 5.3. Geomechanical and Structural Monitoring

#### 5.3.1. Introduction

The geomechanical and structural monitoring of cultural monuments plays a vital role in safeguarding cultural heritage. Geomechanical monitoring of a monument is crucial for ensuring its stability and long-term preservation [33]. Monuments, especially those located in areas with complex geological and engineering geological environments and geotechnical instabilities, are vulnerable to issues such as subsidence and erosion. By continuously monitoring geotechnical parameters such as ground displacements, water table levels, structural and foundation behavior, potential problems can be detected early that may compromise the structure’s stability. This proactive approach allows for timely maintenance or reinforcement measures, preventing severe damage.

Furthermore, geomechanical monitoring provides valuable insights into the interaction between the monument and its surrounding natural environment. Geological formations, underground movements, and changes in groundwater levels can all affect the safety and longevity of the structure. Understanding these factors enables experts to implement long-term management and preservation strategies, helping to protect cultural heritage for future generations.

This paper presents a comprehensive plan for the recording and monitoring of the Agrippa monument’s behavior. Systematic monitoring is essential for the monument’s protection, allowing for the early detection of damages or displacements and thereby ensuring its long-term integrity. The proposed monitoring method integrates modern technologies and methodologies to achieve optimal results in preservation efforts. It includes specific guidelines for the instruments to be utilized and their designated installation locations. Through continuous monitoring, responsible authorities will be equipped to implement timely measures, effectively preventing or minimizing any adverse impacts on this invaluable cultural heritage.

#### 5.3.2. Geomechanical and Structural Monitoring of the Monument

(1) Vertical Displacements

**Figure 17:**
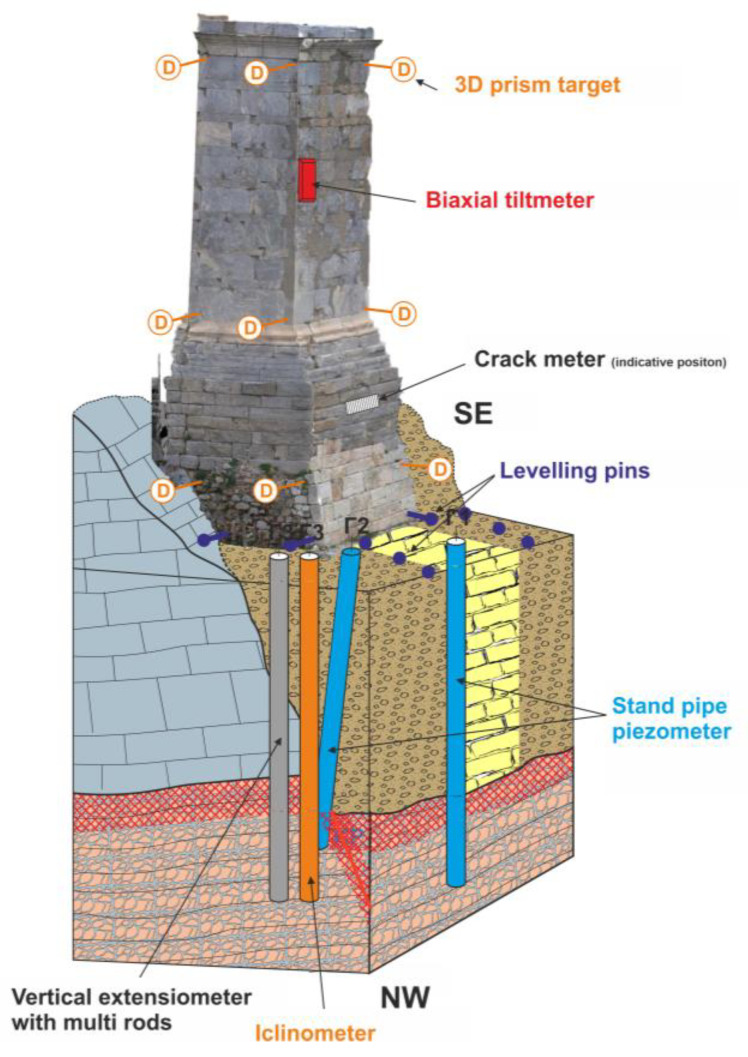
Indicative installation positions of monitoring instruments on the monument and foundation ground.

The monitoring of vertical displacements in the monument’s foundation is crucial for ensuring its structural stability. To achieve this, it is proposed to install levelling pins at strategically selected locations around the base (Figure 17). Specialized surveyors will conduct the measurements using digital levelling instruments, targeting a minimum accuracy of ±1 mm. Frequent measurements will facilitate ongoing monitoring of the foundation’s stability and enable the early detection of any potential anomalies. Measurements should be processed using appropriate survey software, recorded in suitable forms, and analyzed by experienced personnel to ensure accurate evaluation. It is crucial to document any activities on the monument, as well as events such as earthquakes or heavy rainfall, to support reliable assessments. The frequency of levelling pin measurements will depend on the observed movements, ranging from daily in cases of increased activity to monthly when no significant displacements are detected.

(2) 3D Displacement

Monitoring the three-dimensional (3D) movements of the monument includes vertical, horizontal, and longitudinal displacements. The installation of prismatic optical targets at three height positions is proposed, which will allow for systematic monitoring of movements (Figure 17). The 3D targets are proposed to be measured using precision geodetic stations with the free station method. The recommended accuracy for the geodetic stations is ±0.3 mgon (1″) for angles and ±2 mm + 2 ppm for distances. The accuracy of the total 3D displacement measurements will be ±2 mm. Measurements will be conducted by an experienced survey engineer and all measurements should be corrected for the effects of refraction, pressure, and temperature. Data should be processed with appropriate survey software, recorded in suitable forms, and analyzed for easy evaluation. The frequency of 3D target measurements will depend on the displacement rate; if increased, the installation of an automated geodetic station (georobot) is recommended for automatic measurements with a frequency of 30 min. This process will enable the timely detection of risks that may arise from seismic activities, ground changes, or other natural disasters.

(3) Crack openings

The monitoring of cracks opening is equally important. The installation of linear crack meters is proposed, with their selection being determined based on the cracks to be monitored (Figure 17). The crack meters can be either plastic for manual measurements or vibrating wire type for automated measurements. Crack meters can be installed at the foundation and the base of the monument to measure the crack opening in two dimensions, i.e., parallel and perpendicular to the crack direction. Specifically, for each crack selected for monitoring, two crack meters can be placed. One crack meter will measure the crack opening at the installation level (XY), while the second crack meter will measure the crack opening at a level perpendicular to the first one. In the case of choosing automated measurements, vibrating wire crack meters with multiple directions should be installed.

Measurements can be conducted either manually or through automated systems, depending on the monitoring needs. In the case of automatization, the electrical signal from the sensor is collected either at the data logger, where the signal conversion coefficients to mechanical units are applied, or at the data collection box and then transmitted via cables or wireless signal to the digitization box. The conversion coefficients are applied through the automation software, according to the calibration sheet of the sensor. Frequent recording of the cracks will assist in analyzing the behavior of the structural material over time and assessing the need for potential interventions. Due to the importance of the monument, it is proposed to select an automated measurement system with a measurement frequency of every 1 h.

(4) Structural Element Inclination

Monitoring of the inclination of Agrippa’s base is equally indispensable. To this end, the installation of electronic tiltmeters at strategically selected positions is proposed to achieve accurate inclination measurements (Figure 17). The type (uniaxial or biaxial) and location of the tiltmeters will be determined in collaboration with geotechnical and structural engineers. The installation of an electronic tiltmeter provides the option for automated measurements. This monitoring will ensure the timely detection of any anomalies in structural stability, allowing for corrective measures to be taken in real-time.

#### 5.3.3. Geomechanical Monitoring of Ground at the Monument Foundation

(1) Structural Element Inclination

Maintaining the stability and safety of monuments is pivotal and monitoring the ground movements around the monument’s foundation is one of the primary measures to achieve this goal. The proposed monitoring methodology includes the installation of levelling pins at selected locations in the artificial embankments and the bedrock (Figure 17).

A grid with a minimum density of 1.5 m × 1.5 m is proposed to be placed in the surrounding area of the retaining wall, ensuring that significant movements can be detected promptly. The pins are recommended to be made of galvanized steel, measuring 10 cm in length and 1 cm in diameter, featuring a spherical head with a diameter of 3 cm to ensure optimal contact with the ground. Measurements of vertical displacements will be conducted using precision digital instruments, which will provide data with an accuracy of ±1 mm. Measurements should be processed using appropriate survey software, recorded in designated forms, and evaluated by experienced professionals to ensure precision. The frequency of levelling pin measurements will be adjusted according to the detected movements, ranging from daily during heightened activity to monthly when no notable displacements.

(2) Horizontal and vertical displacements with depth

Monitoring both horizontal and vertical ground movements is essential for comprehensively understanding the dynamic forces impacting the monument’s foundation. Τo enhance this monitoring, the installation of an inclinometer and a vertical extensometer is proposed at the northwestern corner of the monument’s base (Figure 17). The inclinometer will be strategically placed to measure any horizontal displacements of the ground, providing critical data on potential tilting or shifting of the foundation. Simultaneously, the vertical extensometer will monitor changes in length or distance between points in the ground, enabling the detection of both horizontal and vertical displacements over time

The installation will occur in newly drilled boreholes, ensuring that the instruments are securely anchored into the bedrock. The depth of these boreholes will be determined based on engineering geological evaluation, ensuring that the instruments can accurately capture data from both the foundation and the underlying strata. The instruments are proposed to be installed at a depth of 3 m into the bedrock to ensure accurate movement measurement and to minimize the effects of surface disturbances. If it is not feasible to drill new boreholes, the installation of an extensometer in the inclined borehole is recommended.

Regular monitoring and data collection from these instruments will be vital. Automated data logging systems may be utilized to provide real-time insights into ground movements, which will facilitate timely responses to any significant changes detected. The recommended frequency for automated measurements is hourly. This proactive approach not only enhances our understanding of the monument’s geomechanical environment but also plays a critical role in the preservation and protection of this invaluable cultural heritage.

(3) Groundwater level fluctuation

The fluctuation of groundwater levels is an important aspect of geomechanical monitoring at the monument foundation. This phenomenon is influenced by various factors, including seasonal changes, precipitation, and nearby water management activities. Regularly monitoring water level variations is significant for understanding their impact on the structural integrity and stability of the monument. To effectively monitor these changes, piezometers must be installed at key positions. Two open-type piezometers are proposed to be installed in drilled boreholes, one beneath the base and one in the surrounding area, to monitor variations in water levels (Figure 17). The piezometers will be made from heavy-duty PVC-U with an outer diameter of 50 mm and will include suitable filters and gravel to ensure proper functioning.

Measurements will be conducted manually using a water level probe. However, if automation is preferred, hydrostatic pressure sensors will be installed to ensure continuous monitoring of groundwater level changes. The integration of automatic sensors within the piezometers will facilitate real-time data collection on water levels, enabling timely interventions in the event of significant variations. By maintaining a comprehensive record of groundwater fluctuations, our ability to assess potential risks and implement effective preservation strategies for the monument is enhanced. The proposed frequency of manual measurements during the winter season is twice per week, while in the summer, it is one measurement every two weeks. In the case of automated piezometer monitoring, measurements will be conducted hourly.

#### 5.3.4. Geomechanical Monitoring Alert and Alarm Limits

An alert limit is defined as a predetermined value or rate of change of a parameter (vertical, horizontal, and longitudinal displacement, angular and horizontal deformation, rate of change of displacement, rate of change of crack opening, etc.), which is considered indicative of a potential impending issue. For the Agrippa monument, it is suggested to set an alert limit for the vertical displacements of the foundation soil after the implementation of the proposed measures, based on total displacements equal to 5 mm.

The alarm limit is defined as the value of aforementioned parameter above which an unacceptable category of damage may occur to the existing and proposed structures. It is suggested to set the alarm limit for vertical displacements of the foundation soil after the implementation of the proposed measures, based on total displacements equal to 8 mm.

## 6. Conclusions

The purpose of this study is to highlight the critical intersection of cultural heritage preservation and geotechnical engineering, particularly in managing uncertainties associated with historical structures. The Agrippa Monument, due to its uneven foundation partially resting on natural bedrock and artificial fills, exemplifies the challenges of assessing and stabilizing ancient monuments with significant lack of information regarding the structural and foundation conditions, stages of construction, and history of remedial measures.

The geotechnical evaluation of the Agrippa Monument’s foundation reveals significant challenges due to the heterogeneous nature of its materials, with artificial backfill on one side and fractured limestone bedrock on the other. This uneven foundation has led to differential settlement and tilting

Through a combination of bibliographic research, geotechnical surveys, and advanced 3D finite element analysis, the study reveals that while the monument does not face immediate risk under current static conditions, several uncertainties persist due to the variability and unknown conditions of the foundation materials, potential water action, and seismic activity. These factors of uncertainty render the reliable prediction of future response of the monument to seismic events practically impossible. To address these concerns, the study recommends specific measures, including grouting to improve the backfill’s properties, controlling water infiltration, and establishing a comprehensive geomechanical and structural monitoring system. By implementing these interventions and closely monitoring the monument’s foundation and structure, the long-term preservation of this invaluable cultural heritage site can be ensured, safeguarding it from potential future deformation and instability.

The results of the study underscore the importance of monitoring, soil improvement, and water management as crucial steps in mitigating future risks. By classifying geomaterials into distinct engineering-geological units and assessing critical parameters, the research contributes to a more nuanced understanding of the foundation’s behavior. Upon application of the proposed measures and installation of the respective monitoring system, the long-term deformational response of the monument to future seismic events can be an interesting field for future research.

The important lesson learned from the specific case study is that for certain examples of very important structures, such as the Agrippa Monument at the Acropolis of Athens, it is much more important and practical to take simple and straightforward action to eliminate the uncertainties and minimize the related risks of a given problem. Exhausting the efforts into trying to approach the “true and accurate” values of all key parameters to maximize the optimization of potential solutions may be a vain struggle.

## Figures and Tables

**Table 1:** Representative range of parameters for ancient, buried wall at the foundation.

Geological Strength Index GSI	Uniaxial Compressive Strength (MPa)	Material Constant m_i_	Disturbance Factor D	Deformation Modulus E_rm_ (MPa)
50	20	7	0.8 ^1^	80–400 ^2^

^1^ Rationally conservative assumption, assuming considerable defects of the wall. ^2^ Application of the most recent methodology by ref. [28] for the estimation of the rock mass deformation modulus by assuming an intact rock modulus value Ei = 3–3.5 GPa, results in values of E_rm_ = 250–320 MPa. The range of parametric analysis was extended as the specific parameter was considered critical.

**Table 2:** Representative range of parameters for artificial deposits.

Effective Cohesion c (kPa)	Friction Angle φ (°)	Unit Weight γ (kN/m^3^)	Poisson’s Ratio v	Deformation Modulus E (MPa)
3–5	26–34 ^1^	20	0.35	8–20 ^1^

^1^ The lowest value of the range is a conservative approach due to the importance of the monument and the uncertainties related to the action of water. The upper bound is a rationally conservative limit given the clay fraction and the low compaction.

**Table 3:** Results of analyses’ group A.

S/N	Parameters of Deposits	Buried Masonry Wall	Base/Foundation of Monument	Settlement on the West Side Static/Seismic (mm)	Maximum Horizontal Movement at Top Static/Seismic (mm)
A1	c = 3 kPa, φ = 26°, E = 8 MPa, v = 0.35	No	Elastic	16–19/22–25	45/66
A2	c = 3 kPa, φ = 26°, E = 8 MPa, v = 0.35	No	Elastic—perfectly plastic	39–58/60–94	136/242
A3	c = 5 kPa, φ = 28°, E = 12 MPa, v = 0.35	No	Elastic—perfectly plastic	24–35/36–57	81/143
A4	c = 5 kPa, φ = 32°, E = 15 MPa, v = 0.35	No	Elastic—perfectly plastic	18–26/26–41	60/104
A5	c = 3 kPa, φ = 26°, E = 8 MPa, v = 0.35	Yes Ε = 400 MPa	Elastic—perfectly plastic	13–18/27–44	47/91
A6	c = 3 kPa, φ = 26°, E = 8 MPa, v = 0.35	Yes Ε = 400 MPa	Plastic with 20% reduction of post peak strength	11–21/20–39	56/173
A7	c = 3 kPa, φ = 26°, E = 8 MPa, v = 0.35	Yes Ε = 100 MPa	Elastic—perfectly plastic	19–23/25–34	58/101
A8	c = 3 kPa, φ = 26°, E = 8 MPa, v = 0.35	Yes Ε = 100 MPa	Plastic with 20% reduction of post peak strength	17–25/25–40	64/173

**Table 4:** Results of analyses’ group B.

S/N	Parameters of Deposits/Buried Masonry Wall	Base/Foundation of Monument	Settlement on the West Side Frequent/Rare Seismic Event (mm)	Maximum Horizontal Movement at Top Frequent/Rare Seismic Event (mm)
B1	c = 3 kPa, φ = 26°, E = 8 MPa, v = 0.35/E = 100 MPa	Elastic	3/4	9/13
B2	c = 3 kPa, φ = 26°, E = 8 MPa, v = 0.35/E = 100 MPa	Elastic—perfectly plastic	22–25/32–37	79/118
B3	c = 3 kPa, φ = 26°, E = 8 MPa, v = 0.35/E = 400 MPa	Elastic—perfectly plastic	13–16/22–28	56/96
B4	c = 50 kPa, φ = 32°, E = 80 MPa, v = 0.35/E = 100 MPa	Elastic	2/3	8/11
B5	c = 50 kPa, φ = 32°, E = 80 MPa, v = 0.35/E = 100 MPa	Elastic—perfectly plastic	5–7/7–8	20/27
B6	c = 50 kPa, φ = 32°, E = 80 MPa, v = 0.35/E = 400 MPa	Elastic—perfectly plastic	3–4/4–5	13/19

## Data Availability

Data are contained within the article.
